# Supplementary material for: Measuring multi-year changes in the Symbiodiniaceae algae in Caribbean corals on coral-depleted reefs
Source: PeerJ. 2024 May 21;12:e17358. doi: 10.7717/peerj.17358 (PMC11141555; doi:10.7717/peerj.17358)
Supplement: Supplemental Information 1 [file peerj-12-17358-s001.docx]

Supplementary Information for

**Measuring multi-year changes in the Symbiodiniaceae algae in Caribbean corals on coral-depleted reefs**

Ross Cunning^1^, Elizabeth A. Lenz^2^, Ruth D. Gates^3*^, Peter J. Edmunds^4^

1 Daniel P. Haerther Center for Conservation and Research, John G. Shedd

Aquarium, 1200 South Lake Shore Drive, Chicago, IL 60605, USA

2 University of Hawaiʻi Sea Grant College Program, University of Hawai‘i at

Mānoa, 2525 Correa Rd Honolulu, HI, 96822, USA

3 Hawai’i Institute of Marine Biology, University of Hawai’i at Mānoa, 46-007

Lilipuna Rd Kāneʻohe, HI, 96744, USA

4 Department of Biology, California State University, 18111 Nordhoff Street,

Northridge, CA 91330-8303, USA

* Deceased

**Supplementary Figures and Tables**


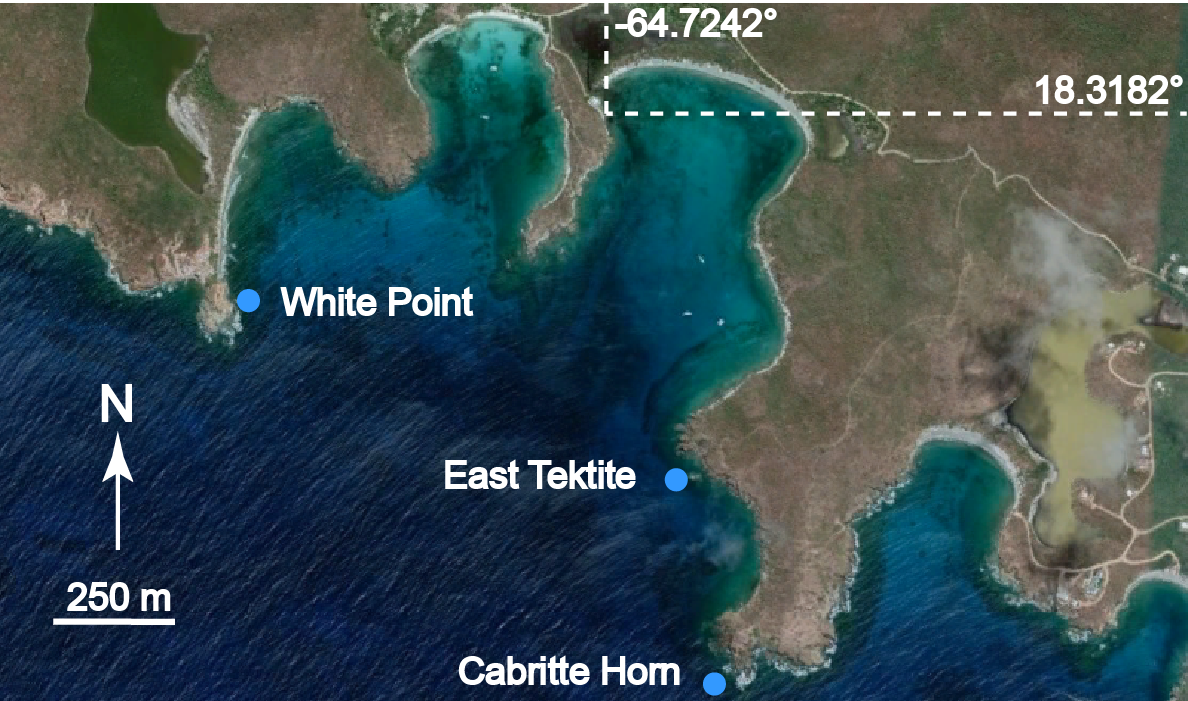


Map data ©2024 Google

**Figure S1.** Aerial image of the south shore of St. John showing the three study sites, White point, East Tektite, and Cabritte Horn. Dashed lines show latitude and longitude of the laboratory dock from which the fieldwork was staged. Map data ©2024 Google.

**Table S1.** Pairwise PERMANOVA tests for differences in composition of *Breviolum* sequence variants between host species. P-values were adjusted using the Benjamini-Hochberg method.

| **pairs** | **Df** | **SumsOfSqs** | **F.Model** | **R2** | **p.value** | **p.adjusted** | **sig** |
| --- | --- | --- | --- | --- | --- | --- | --- |
| franksi vs cavernosa | 1 | 0.79942217 | 2.95139717 | 0.37117969 | 0.028 | 0.042 | . |
| franksi vs siderea | 1 | 0.35606032 | 0.96830393 | 0.24400952 | 0.4 | 0.4 |  |
| franksi vs strigosa | 1 | 0.45989451 | 2.24477147 | 0.11088154 | 0.053 | 0.07227273 |  |
| franksi vs natans | 1 | 0.77125938 | 3.84385387 | 0.17596958 | 0.023 | 0.03833333 | . |
| franksi vs labrynthiformis | 1 | 0.75766371 | 4.64417294 | 0.17430351 | 0.003 | 0.00642857 | * |
| cavernosa vs siderea | 1 | 1.04683163 | 6.92700342 | 0.63393441 | 0.06666667 | 0.08333333 |  |
| cavernosa vs strigosa | 1 | 2.22491103 | 13.2556755 | 0.41095638 | 0.001 | 0.0025 | * |
| cavernosa vs natans | 1 | 2.28688118 | 13.9578335 | 0.4235058 | 0.001 | 0.0025 | * |
| cavernosa vs labrynthiformis | 1 | 2.46808419 | 18.3679404 | 0.4440139 | 0.001 | 0.0025 | * |
| siderea vs strigosa | 1 | 0.21500379 | 1.2441047 | 0.06819215 | 0.235 | 0.27115385 |  |
| siderea vs natans | 1 | 1.24926158 | 7.4209182 | 0.30387548 | 0.013 | 0.024375 | . |
| siderea vs labrynthiformis | 1 | 0.14720085 | 1.08871576 | 0.04928832 | 0.318 | 0.34071429 |  |
| strigosa vs natans | 1 | 5.13199301 | 30.1526391 | 0.48513852 | 0.001 | 0.0025 | * |
| strigosa vs labrynthiformis | 1 | 1.15190092 | 7.6454951 | 0.17517261 | 0.001 | 0.0025 | * |
| natans vs labrynthiformis | 1 | 5.8302681 | 39.2476131 | 0.52157951 | 0.001 | 0.0025 | * |


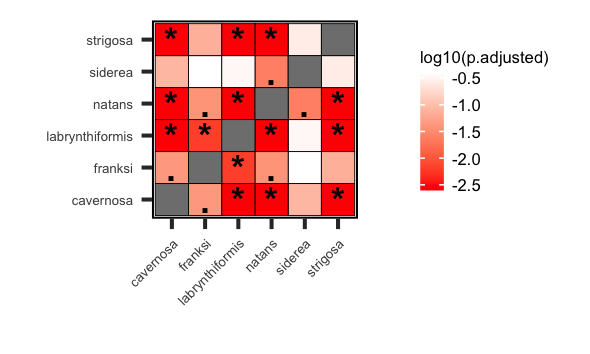


**Figure S2.** Heatmap showing pairwise comparisons (by PERMANOVA) in the composition of *Breviolum* sequences variants hosted by different coral species. Asterisks indicate adjusted p < 0.01, while periods indicate p < 0.05.

**Table S2.** Pairwise PERMANOVA tests for differences in composition of *Cladocopium* sequences between host species. P-values were adjusted by the Benjamini-Hochberg method.

| **pairs** | **Df** | **SumsOfSqs** | **F.Model** | **R2** | **p.value** | **p.adjusted** | **sig** |
| --- | --- | --- | --- | --- | --- | --- | --- |
| siderea vs faveolata | 1 | 5.92672023 | 111.698332 | 0.7827585 | 0.001 | 0.002 | * |
| siderea vs annularis | 1 | 5.33999542 | 75.4650298 | 0.71554552 | 0.001 | 0.002 | * |
| siderea vs franksi | 1 | 6.07694524 | 116.890054 | 0.79038482 | 0.001 | 0.002 | * |
| siderea vs cavernosa | 1 | 2.31164792 | 22.9082051 | 0.34757744 | 0.001 | 0.002 | * |
| siderea vs natans | 1 | 2.98742604 | 52.6742832 | 0.66112026 | 0.001 | 0.002 | * |
| siderea vs strigosa | 1 | 2.55418658 | 47.1185759 | 0.64441321 | 0.001 | 0.002 | * |
| siderea vs furcata | 1 | 2.54616633 | 31.2672399 | 0.55569173 | 0.001 | 0.002 | * |
| siderea vs labrynthiformis | 1 | 1.44960441 | 25.2604256 | 0.52341904 | 0.008 | 0.01107692 | . |
| faveolata vs annularis | 1 | 0.12498733 | 1.73426136 | 0.09257164 | 0.123 | 0.12651429 |  |
| faveolata vs franksi | 1 | 0.10239761 | 2.58148565 | 0.12542757 | 0.053 | 0.06154839 |  |
| faveolata vs cavernosa | 1 | 5.15598562 | 44.9461791 | 0.59971275 | 0.001 | 0.002 | * |
| faveolata vs natans | 1 | 3.2346539 | 71.4668459 | 0.83619379 | 0.001 | 0.002 | * |
| faveolata vs strigosa | 1 | 2.91788559 | 74.1239913 | 0.85078737 | 0.001 | 0.002 | * |
| faveolata vs furcata | 1 | 2.18935012 | 23.0831389 | 0.65795535 | 0.002 | 0.0036 | * |
| faveolata vs labrynthiformis | 1 | 1.47909556 | 35.030374 | 0.77792767 | 0.016 | 0.02057143 | . |
| annularis vs franksi | 1 | 0.16965587 | 2.41966046 | 0.12459849 | 0.072 | 0.07623529 |  |
| annularis vs cavernosa | 1 | 4.66602369 | 34.5242168 | 0.54348119 | 0.001 | 0.002 | * |
| annularis vs natans | 1 | 3.01660007 | 35.277857 | 0.73072541 | 0.001 | 0.002 | * |
| annularis vs strigosa | 1 | 2.73986567 | 33.2199575 | 0.73463045 | 0.002 | 0.0036 | * |
| annularis vs furcata | 1 | 1.96416325 | 13.3688528 | 0.54860411 | 0.003 | 0.00469565 | * |
| annularis vs labrynthiformis | 1 | 1.40746282 | 14.0714045 | 0.60990671 | 0.026 | 0.03227586 | . |
| franksi vs cavernosa | 1 | 5.32226769 | 46.8478799 | 0.60961838 | 0.001 | 0.002 | * |
| franksi vs natans | 1 | 3.31599964 | 77.3171377 | 0.84668814 | 0.001 | 0.002 | * |
| franksi vs strigosa | 1 | 2.9932166 | 81.3157417 | 0.86216511 | 0.001 | 0.002 | * |
| franksi vs furcata | 1 | 2.19757974 | 23.8664391 | 0.66542539 | 0.003 | 0.00469565 | * |
| franksi vs labrynthiformis | 1 | 1.51369187 | 38.9108162 | 0.79554625 | 0.012 | 0.016 | . |
| cavernosa vs natans | 1 | 1.96410687 | 15.3451152 | 0.37114699 | 0.001 | 0.002 | * |
| cavernosa vs strigosa | 1 | 1.56090309 | 12.1718228 | 0.32744756 | 0.001 | 0.002 | * |
| cavernosa vs furcata | 1 | 2.24407663 | 14.0533372 | 0.3693063 | 0.001 | 0.002 | * |
| cavernosa vs labrynthiformis | 1 | 1.19463765 | 8.4332829 | 0.27710724 | 0.003 | 0.00469565 | * |
| natans vs strigosa | 1 | 0.01516977 | 0.342872 | 0.03669878 | 0.861 | 0.861 |  |
| natans vs furcata | 1 | 1.82540253 | 14.2525961 | 0.64049139 | 0.008 | 0.01107692 | . |
| natans vs labrynthiformis | 1 | 1.17584219 | 22.8557396 | 0.7920691 | 0.044 | 0.0528 |  |
| strigosa vs furcata | 1 | 1.72451471 | 13.3728901 | 0.65640614 | 0.005 | 0.0075 | * |
| strigosa vs labrynthiformis | 1 | 1.11663765 | 29.893555 | 0.85670706 | 0.057 | 0.064125 |  |
| furcata vs labrynthiformis | 1 | 1.04038217 | 5.11759592 | 0.56128786 | 0.06666667 | 0.07272727 |  |


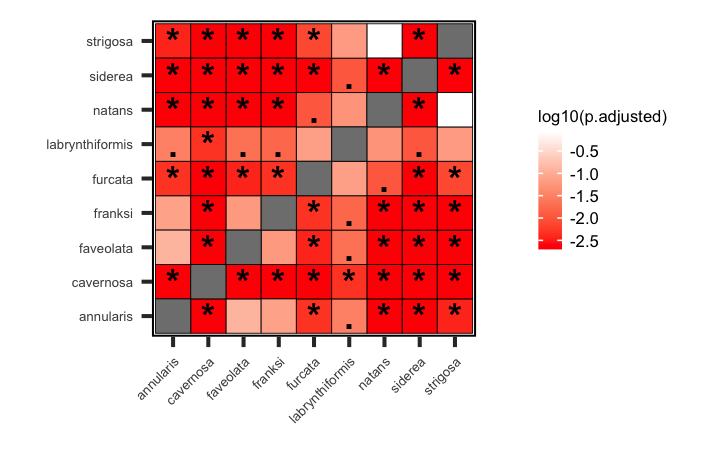


**Figure S3.** Heatmap showing pairwise comparisons (by PERMANOVA) in the composition of *Cladocopium* sequences variants hosted by different coral species. Asterisks indicate adjusted p < 0.01, while periods indicate p < 0.05.
